# Insights into geometric deviations of medical 3d-printing: a phantom study utilizing error propagation analysis

**DOI:** 10.1186/s41205-024-00242-x

**Published:** 2024-11-22

**Authors:** Lukas Juergensen, Robert Rischen, Julian Hasselmann, Max Toennemann, Arne Pollmanns, Georg Gosheger, Martin Schulze

**Affiliations:** 1 Department of General Orthopedics and Tumor Orthopedics, University Hospital Muenster, Münster, 48149 Germany; 2 Clinic for Radiology, University Hospital Muenster, Muenster, 48149 Germany; 3 Materials Engineering Laboratory, Department of Mechanical Engineering, University of Applied Sciences Muenster, Steinburg, 48565 Germany

**Keywords:** Quality assurance, Medical 3D-printing, Segmentation error, Digital editing error, Printing error, Patient specific, Effect of kernel, Effect of slice thickness, Effect of smooting, FFF-3D-printing, Warping, Point-of-care

## Abstract

**Background:**

The use of 3D-printing in medicine requires a context-specific quality assurance program to ensure patient safety. The process of medical 3D-printing involves several steps, each of which might be prone to its own set of errors. The segmentation error (SegE), the digital editing error (DEE) and the printing error (PrE) are the most important partial errors. Approaches to evaluate these have not yet been implemented in a joint concept. Consequently, information on the stability of the overall process is often lacking and possible process optimizations are difficult to implement. In this study, SegE, DEE, and PrE are evaluated individually, and error propagation is used to examine the cumulative effect of the partial errors.

**Methods:**

The partial errors were analyzed employing surface deviation analyses. The effects of slice thickness, kernel, threshold, software and printers were investigated. The total error was calculated as the sum of SegE, DEE and PrE.

**Results:**

The higher the threshold value was chosen, the smaller were the segmentation results. The deviation values varied more when the CT slices were thicker and when the threshold was more distant from a value of around -400 HU. Bone kernel-based segmentations were prone to artifact formation. The relative reduction in STL file size [as a proy for model complexity] was greater for higher levels of smoothing and thinner slice thickness of the DICOM datasets. The slice thickness had a minor effect on the surface deviation caused by smoothing, but it was affected by the level of smoothing. The PrE was mainly influenced by the adhesion of the printed part to the build plate. Based on the experiments, the total error was calculated for an optimal and a worst-case parameter configuration. Deviations of 0.0093 mm ± 0.2265 mm and 0.3494 mm ± 0.8001 mm were calculated for the total error.

**Conclusions:**

Various parameters affecting geometric deviations in medical 3D-printing were analyzed. Especially, soft reconstruction kernels seem to be advantageous for segmentation. The concept of error propagation can contribute to a better understanding of the process specific errors and enable future analytical approaches to calculate the total error based on process parameters.

**Supplementary Information:**

The online version contains supplementary material available at 10.1186/s41205-024-00242-x.

## Background

### 3D-printing in medicine

Medical 3D-printing continues to transform the way patients are treated. It can be used to create anatomical models [1, 2], patient-specific instruments (PSI) [3, 4] and custom made implants [5, 6], enabling fast, accurate, and personalized treatment approaches. This technological advancement aids in patient information [7, 8] as well as medical training [9, 10] and contributes to the success of surgeries and procedures [11, 12]. However, the effective and safe use of 3D-printing in medicine requires a rigorous and context-specific quality assurance (QA) program to ensure a stable production process in terms of patient safety.

### The medical 3D-printing process and its errors

The process of medical 3D-printing is complex and involves several steps, each of which might be prone to its own set of errors. It begins with imaging and then proceeds with segmentation, which generates virtual 3D-models from Digital Imaging and Communications in Medicine (DICOM) datasets. Typically, these models are saved as Standard Tessellation Language (STL) files, which represent the model as a 3D mesh of triangles and vectors. However, STL files can contain errors such as artefacts, mesh gaps, and vector misalignments. These issues can be addressed through a variety of automated and manual repair methods, such as smoothing or vector correction. These digital editing techniques are critical to create a high-quality, printable STL file, which is then converted into a code that can be processed by the 3D-printer to build an object layer by layer (slicing). Once the build has been completed, depending on the printing technology employed, material-specific post-processing steps may be required, such as the removal of support structures.

**Fig. 1:**
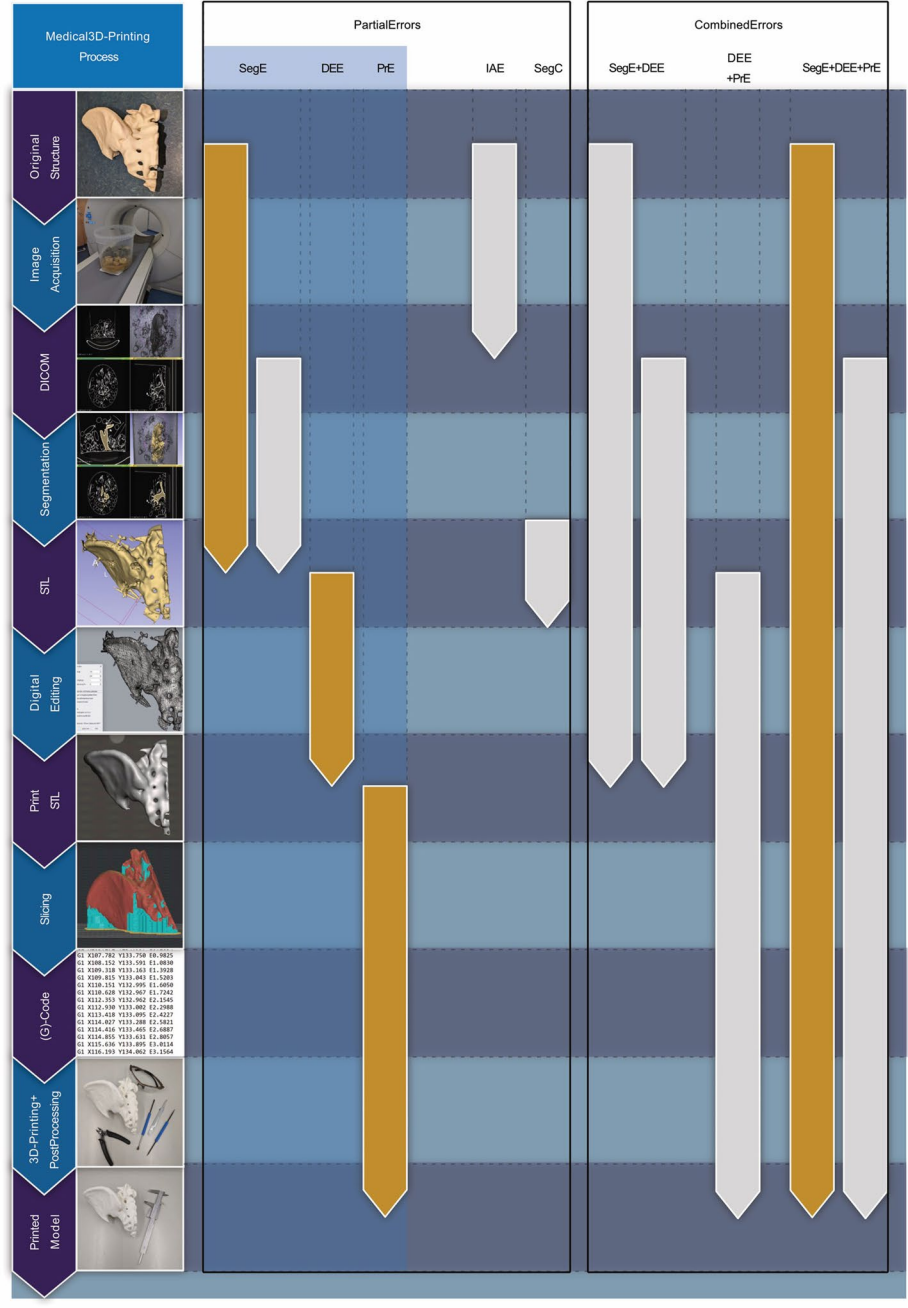
The medical 3D-printing process, its main types of error and their possible combinations (according to Schulze et al. [13]. Highlighted with orange: focus of this study. The arrows indicate which of the intermediate process results are compared to determine the respective main types of error, including the differentiation of *combined* and *partial errors*. Highlighted with blue box: Errors that should be evaluated individually according to the guidelines of the RSNA for medical 3D-printing [14]. SegE: *Segmentation Error*, DEE: *Digital Editing Error*, PrE: *Printing Error*, IAE: *Image Acquisition Error*, SegC: *Segmentation Comparison Error*

A terminology for errors in medical 3D-printing has been introduced in a recent review on quality assurance for 3D-printed patient specific anatomical models by Schulze et al. [13]. It distinguishes the three most important partial errors (segmentation error (SegE), digital editing error (DEE) and printing error (PrE)) and their possible combinations. According to the definitions by Schulze et al., “the SegE is defined as the deviation between the original structure and the direct result of the segmentation process”, “the DEE is defined as the deviation between the direct result of the segmentation process and the print-STL” and “the PrE is defined as the deviation between the print-STL and the printed model”. Figure 1 shows the process to produce patient specific 3D-printed anatomical models, the main types of errors that can occur, their possible combinations and the focus of this study.

### Quality assurance for medical 3D-printing

The challenges of achieving reliable segmentation results have been described in various contexts [15–20]. For the most part, they can be attributed to the often-required significant manual intervention during segmentation, but parameters such as slice thickness, image reconstruction algorithm and threshold can also affect the segmentation results. In contrast to the SegE, the subsequent process steps (DEE and PrE) are less dependent on manual intervention and primarily influenced by software parameters. While several studies focused on parameters, that have an impact on the SegE and PrE, the DEE has not yet been comprehensively investigated. Many studies addressed only the quality of the results, comparing the original structures with the final printed product [13]. However, those approaches have not yet been implemented in a joint concept and the latter often lack information on the stability of the process and possible optimizations.

With regard to the SegE, there is a lack of literature on the influence of the CT reconstruction parameter "kernel" on the accuracy of the segmentation results. In general, the choice of reconstruction kernel is a trade-off between resolution and noise. Hard kernels provide better sharpness of edges but have a lower signal-to-noise ratio compared to softer kernels [21–23]. Since a high signal-to-noise ratio can greatly simplify the segmentation process, it is questionable whether soft kernels should generally be preferred for segmentation purposes for 3D-printing, even when the target structure has a high contrast to the surrounding tissue (e.g., segmenting bones).

In accordance with the recommendations of the Radiological Society of North America on quality assurance for medical 3D-printing by Chepelev et al., this study evaluates the SegE, DEE and PrE individually [14]. Ten different configurations of CT imaging parameters, seven different threshold settings, three software variations and smoothing levels as well as three fused filament fabrication (FFF) 3D-printers are considered.

Among the software variants, Brainlab Elements has not yet been frequently examined in terms of segmentation for 3D-printing. However, performing segmentation with Brainlab Elements enables an efficient combination of 3D-printing and navigation [24].

Finally, an exemplary concept based on error propagation is introduced to analyze the cumulative effect of the partial errors, thereby enabling a comprehensive assessment of their influences on the total error. Based on this, the key learnings for the process owner and clinical user are summarized in Fig. 12.

## Materials and Methods

**Fig. 2:**
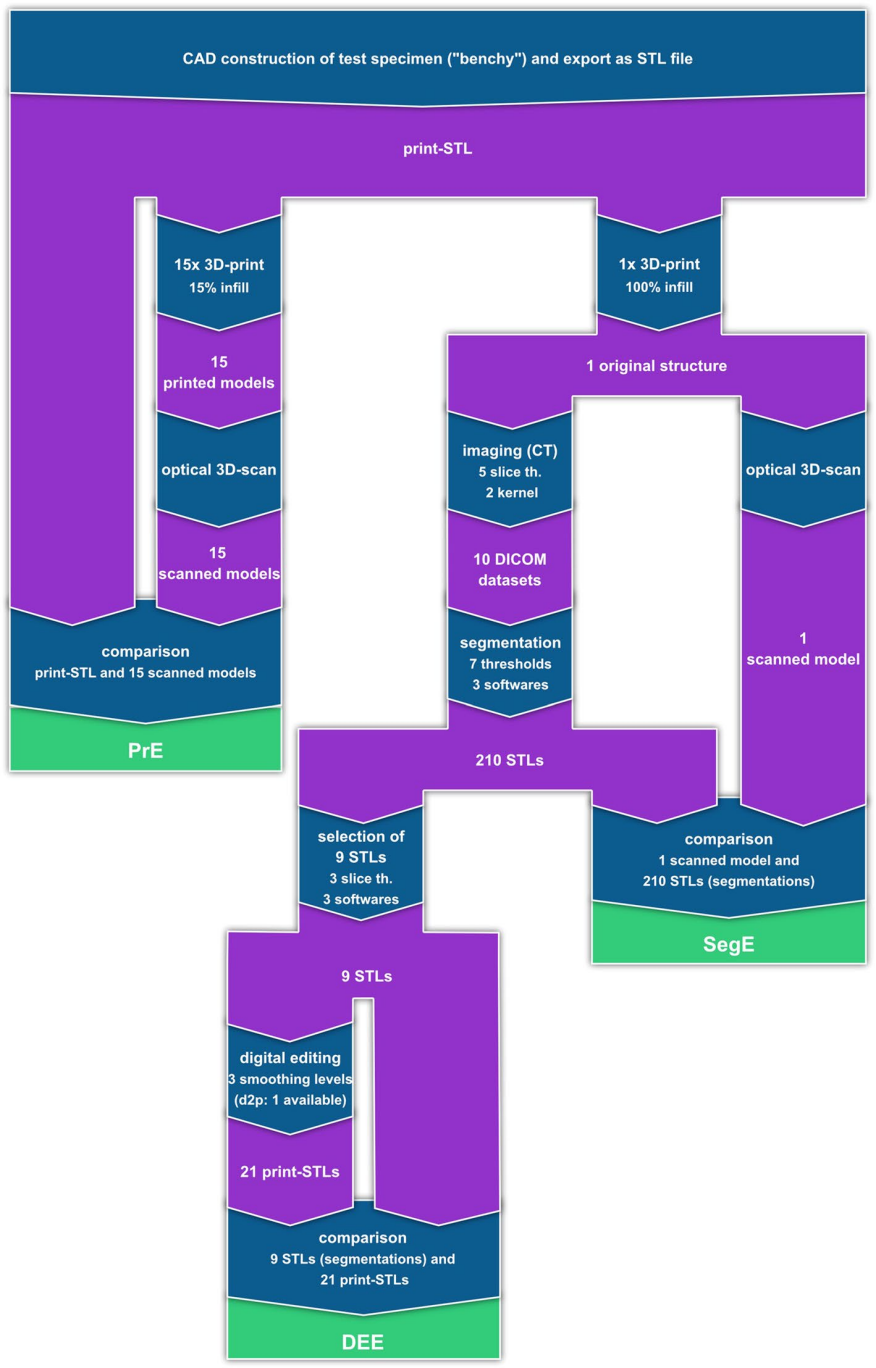
Summary of the study protocol. The SegE, DEE and PrE are evaluated individually according to the definitions by Schulze et al. [13]. th.: thickness

A summary of the study protocol is visualized in Fig. 2 and a detailed description of the methods is provided in the following sections. To begin with, a test specimen was designed using CAD software (Fusion360, Autodesk, San Rafael, USA). The test specimen is also referred to as the "benchy" in this study and was used for all experiments.

### Production of the phantom

Generally, an artificial phantom has three main advantages over real anatomical structures for the evaluation of a medical 3D printing process:

1. It resembles real anatomy without the issues of individual structural variations, privacy concerns, and shareability.
2. It enables the structured representation of typical anatomical structure correlates (large and small curves, corners, different angles, etc.), which improves universal transferability.
3. It can be printed without support structures. As support structures are generated by the slicing tool and can affect the quality of surface, the determined printing error is less influenced by the slicing tool when no support structures are required. 

To enable a broad application of our concept, the STL-file of the benchy is shared with the supplementary materials in the online version of this article.

**Fig. 3:**
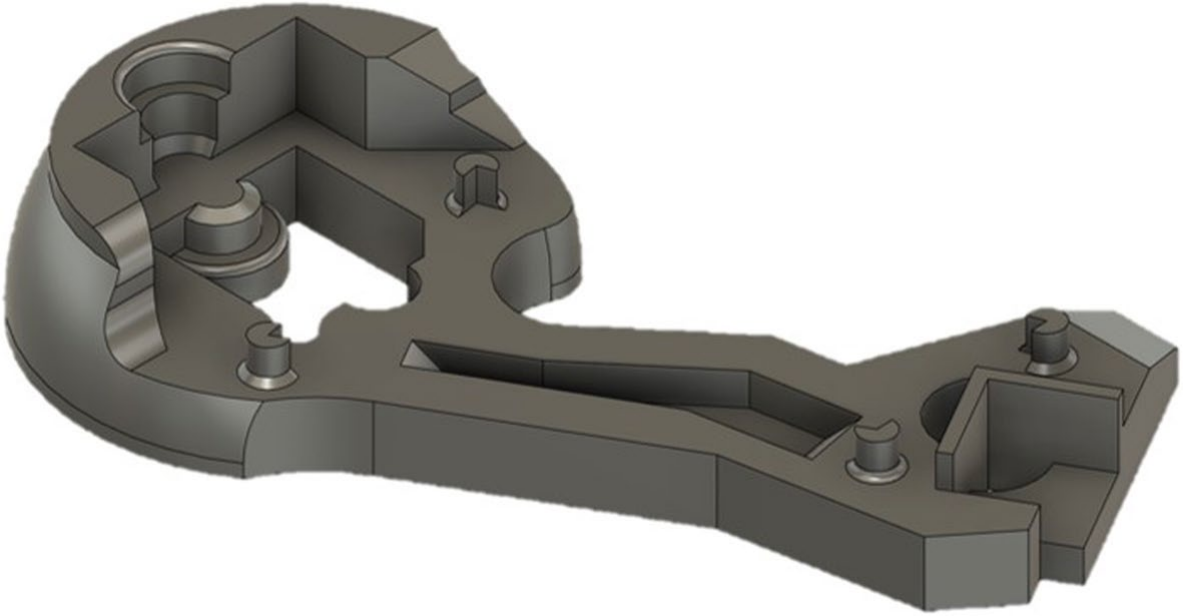
Benchy

The benchy mimics the shape of the proximal femur and is specifically designed to simulate the challenges of segmentation. It includes freeform surfaces and distinct areas to evaluate the impact of various sources of error on differently shaped structures. The benchy is shown in Fig. 3 and the STL file is online available in the supplementary materials. After construction, the benchy was exported as a STL file and then 3D-printed in poly lactid acid (PLA) using a Raise3D Pro2 printer (Raise3D, Shanghai, China) with 100% infill, a slice thickness of 0.3 mm and a nozzle diameter of 0.6 mm.

### Image acquistion

**Fig. 4:**
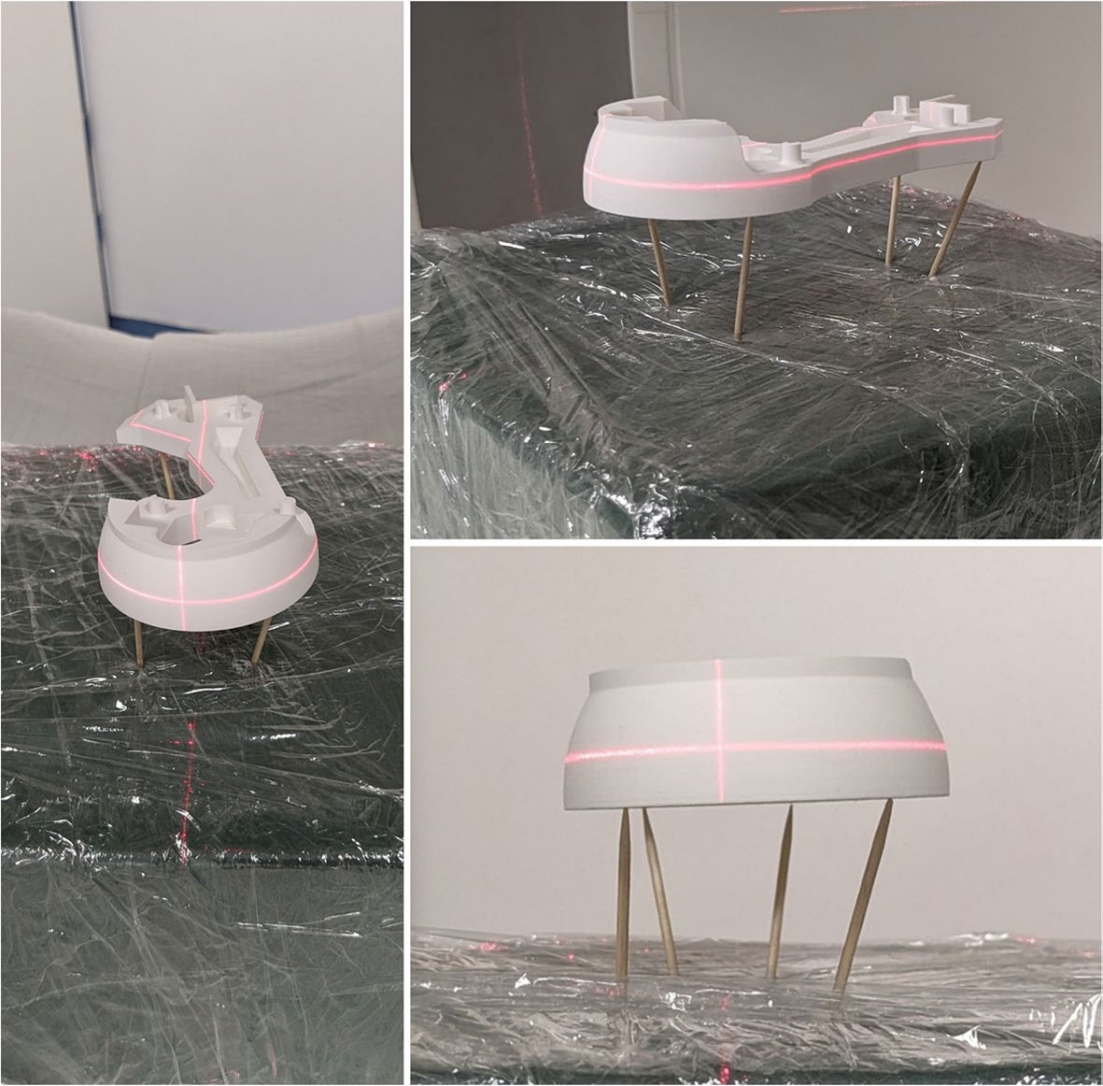
Experimental setup of the CT image acquisition

Ten computed tomography (CT) scans (Somatom Definition Flash, Siemens, Germany) of the benchy were acquired employing ten different parameter combinations. Five scans with slice thicknesses of 0.4, 0.6, 1.5, 3.0, and 5.0 mm were acquired with each of one soft tissue (STK) and one bone kernel (BK). Common standard kernels for soft tissue and bone tissue were selected according to the protocols with the respective slice thicknesses as defined for clinical use. Tube voltage (kV) and tube current (mAs) were adopted according to the default settings of the protocols (Table 1, Appendix A). To minimize the contact between the benchy and its mount during imaging, it was positioned on four toothpicks. Figure 4 shows the experimental setup of the CT image acquisition.

### Evaluation of the SegE

The benchy was 3D-scannend with an optical 3D-scanner (GOM Atos Core, GOM, Germany, accuracy up to 0.03 mm) to generate a virtual ground truth 3D-model as a reference. Threshold segmentations of the benchy were performed on each of the ten DICOM datasets using the three different software programs 3D Slicer (Version 5.0.3, Brigham and Women’s Hospital, Harvard Medical School, USA), D2P (Version 1.0.5, 3D Systems, USA), and Brainlab Elements (Origin server 3.3., Brainlab, Germany) with threshold settings increasing incrementally in seven steps from -800 to 200 HU. Only minimal manual intervention was required to remove the toothpicks from the segmentation.

Finally, the resulting 210 benchy segmentations were exported as STL files and compared to the ground truth 3D-scan model by surface deviation analysis after automatic alignment minimizing the total deviation using GOM Inspect (2022, Service Pack1, GOM, Germany).

### Evaluation of the DEE

Three of the resulting segmentations of each software (based on bone kernel DICOM data sets with slice thicknesses 0.4 mm, 1.5 mm, 5.0 mm; threshold of -600 HU) were selected to evaluate the DEE. These segmentations were further processed applying low, medium and strong smoothing. In D2P only a low smoothing option was available. Since there is no uniform scale for defining the degree of smoothing, settings were chosen based on the visual impression of an experienced examiner, which were classified as low, medium and high. The medium category most closely approximates a setting that would have been chosen for clinical use. An attempt was made to select similar parameters for all three software variants; however, the absolute error values of the DEE are still not directly comparable between the software because the level of smoothing is defined by a different scale in each software. For the category "low", a median smoothing with a kernel size of 1 mm was chosen in 3D Slicer, a smoothing factor of 0.66 in Brainlab Elements, and a factor of 1 mm in D2P. For the category "medium", a median smoothing with a kernel size of 3 mm was selected in 3D Slicer and a smoothing factor of 1.33 in Brainlab Elements. For the category "high", a median smoothing with a kernel size of 5 mm and a smoothing factor of 2.00 were selected in 3D Slicer and Brainlab Elements, respectively. The resulting 21 print-STLs were compared to the corresponding direct segmentation results by surface deviation analysis after automatic alignment minimizing the total deviation using GOM Inspect.

### Evaluation of the PrE

**Fig. 5:**
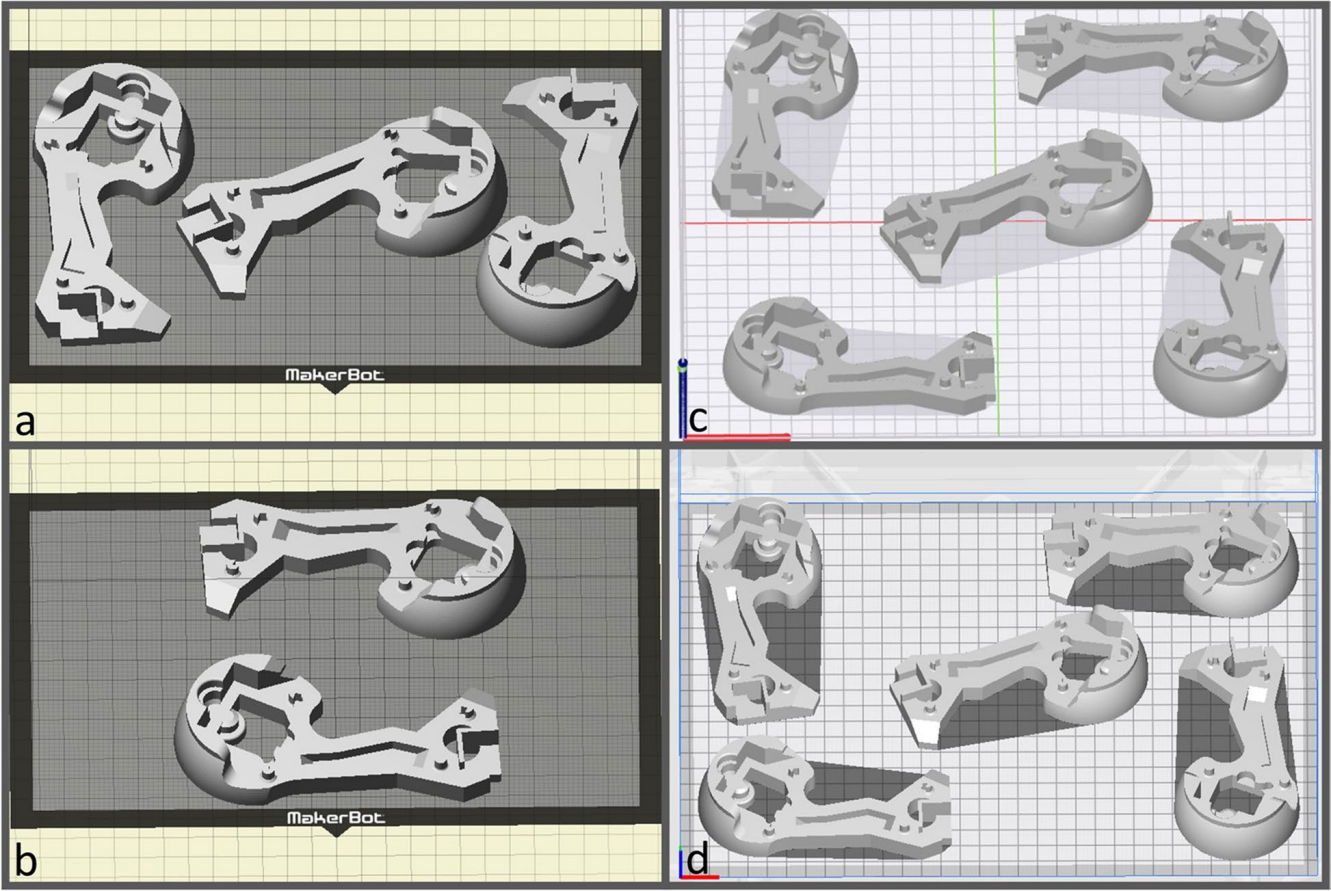
Print bed orientations and positions of the 15 printed benchys for the evaluation of the PrE. a, b: Replicator 2X, c: Ultimaker S5, d: Raise3D Pro2

In addition to the benchy that was printed with 100% infill, 15 further benchys were printed using three different FFF 3D-printers (Replicator 2X, Makerbot, New York, USA; Ultimaker S5, Ultimaker, Utrecht, Netherlands; RaisePro2, Raise3D. Shanghai, China). Five benchys were printed with each of the three printers with different print bed positions and orientations. The layer thickness and infill were kept constant at 0.3 mm and 15%. The nozzle diameters were 0.4 mm, 0.6 mm and 0.8 mm for the Replicator 2X, the Raise2pro and Ultimaker S5. Figure 5 shows the print positions and orientations for all printers. To evaluate the printer-specific PrE the printed benchy models were 3D-scannend with an optical 3D-scanner (GOM Atos Core) and compared to the construction STL by surface deviation analysis after automatic alignment minimizing the total deviation using GOM Inspect.

### Statistical characterization of surface deviation analyses

To examine SegE, DEE and PrE, surface deviation analyses were performed using GOM Inspect. The results of the surface deviation analyses were then exported as ASCII (American Standard Code for Information Interchange) files and analyzed with Matlab (R2023b, Mathworks, USA). The mean surface deviation was used as a measure for the position of the deviation values. This specifically provides information on whether the analyzed object is larger or smaller than the reference (negative mean values indicate that the analyzed object is smaller than the reference). The 6σ-interval provides information on the variation of the deviation values. The 6σ-interval is the size of the interval of the 3-σ-environment and was defined as the difference between $mean+3\sigma$ and $mean-3\sigma$. For normally distributed values, 99.7% of the values fall within this interval.

### Evaluation of the Total Error

The total error was calculated as the sum of SegE, DEE and PrE according to the rules of the Gaussian error propagation as shown in Eq. 1 and 2 [25]. ${\Delta mean}_{total}$ represents the mean surface deviation of the total error and ${\sigma }_{total}$ its standard deviation.

1 $${\Delta mean}_{Total}={\Delta mean}_{SegE}+{\Delta mean}_{DEE}+{\Delta mean}_{PrE}$$

2 $${\sigma }_{Total}=\sqrt{{\sigma }_{SegE}^{2}+{\sigma }_{DEE}^{2}+{\sigma }_{PrE}^{2}}$$

The total error has been calculated as an example for two constellations of parameters that have been determined in this study. The calculation was based on the results of 3D Slicer. In both cases, the data was derived from STK segmentations. The average of the five Ultimaker S5 prints was considered for the PrE in both cases.

## Results

### Evaluation of the SegE

**Fig. 6:**
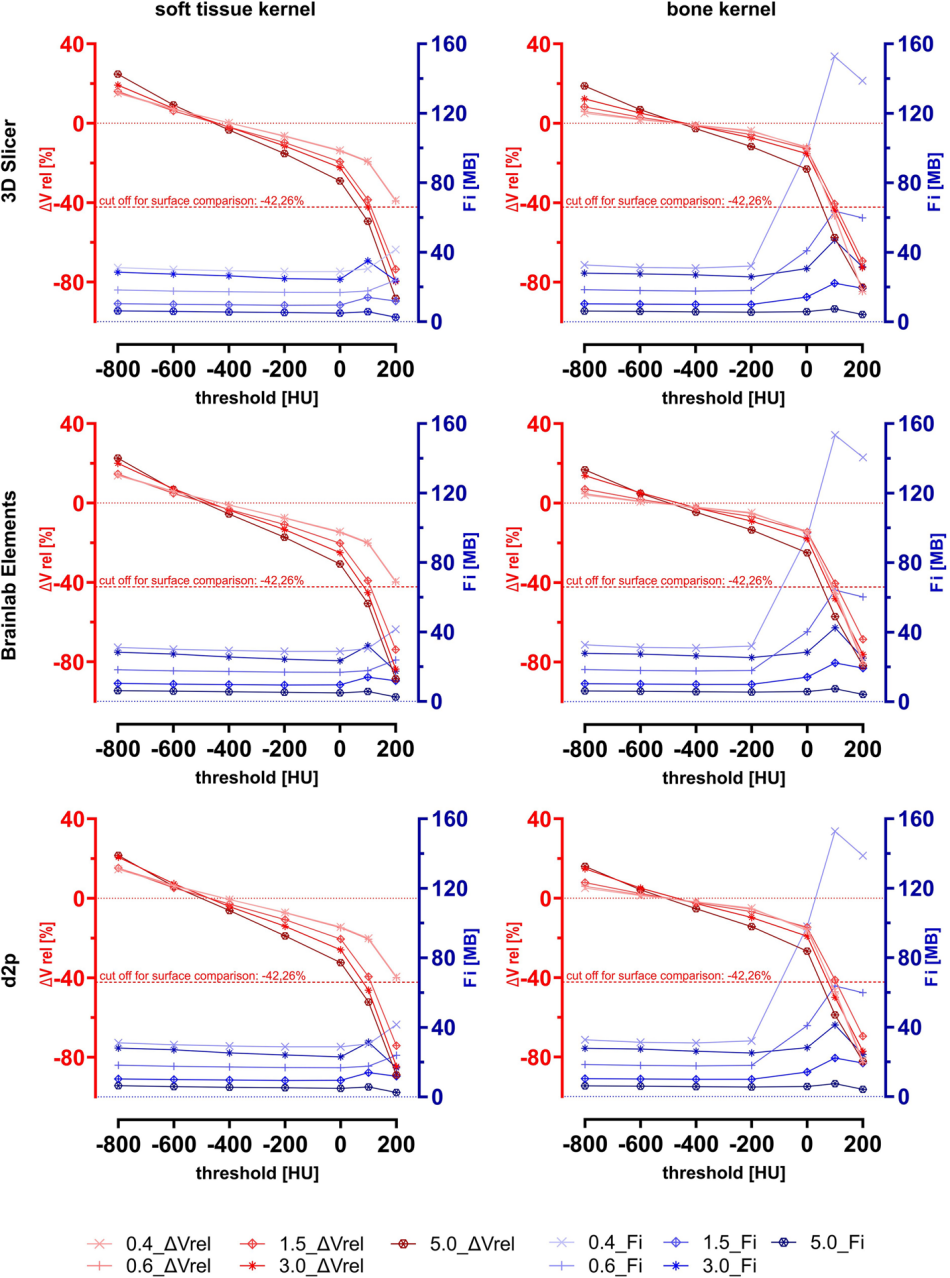
Relative volume errors of the segmentation process (∆V rel) and the file sizes (Fi; MB: megabyte) of the segmentation results. Each of the five red and blue lines in each diagram reflect the CT with corresponding slice thickness of 0.4 mm, 0.6 mm, 1.5 mm, 3.0 mm and 5.0 mm

**Fig. 7:**
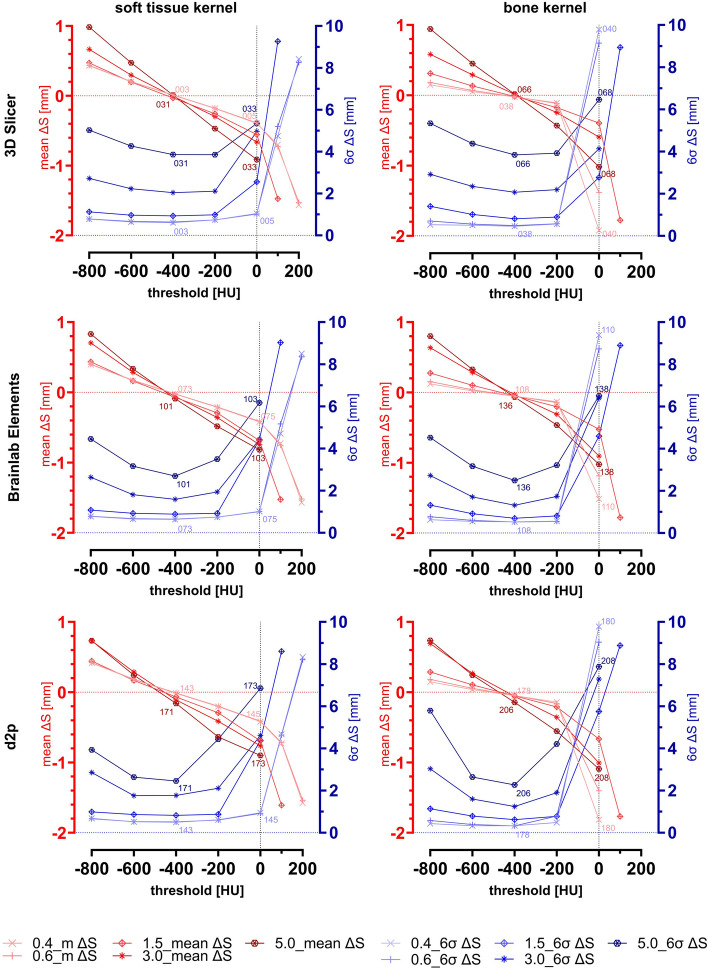
Means (mean ∆S) and 6σ-intervals (6σ ∆S) of the surface deviation between reference 3D-scan and direct segmentation results. Each of the five red and blue lines in each diagram reflect the CT with corresponding slice thickness of 0.4 mm, 0.6 mm, 1.5 mm, 3.0 mm and 5.0 mm. A detailed analysis for the segmentations highlighted with numbers is provided in Figure 13-Figure 16 of Appendix A

The relative error of volume caused by segmentation (negative values indicate that the volume of the segmentation result is lower than the volume of the ground truth 3D-scan) is shown in Fig. 6 together with the file size (Fi) of the segmentation results. The parameter Fi reflects the complexity of the surface of the segmentation results as the Fi of a STL file is proportional to the number of polygons it contains. Each line in the diagrams reflects a layer thickness of the corresponding CT DICOM data sets (0.4, 0.6, 1.5, 3.0, and 5.0 mm). The X-axis indicates the threshold chosen for segmentation in Hounsfield Units (HU). Additionally, the kernel and the software used are differentiated. When the differences between the surfaces of the reference and segmentation results became too large, the iterative closest point alignment in GOM Inspect could no longer be performed reliably. As a consequence, it was not possible to analyze the surface deviation for these samples. Therefore, a cut-off value was defined for the volume error. Exceeding this value, surface deviation analysis was not possible. The volume of the reference 3D-scan of the printed benchys was 51,955 mm^3^. If the volume of the segmentation result was less than 30,000 mm^3^, no surface deviation analysis was performed. This corresponds to a relative volume error of -42.26%. This threshold is also shown in Fig. 6. For all cases with a relative volume error exceeding the cut-off of -42.26%, no values are plotted in Fig. 7, as the mean and the 6σ-interval of the surface deviation could not be calculated for these cases.

In summary, the file size of the segmentation result showed a peak around a threshold of 100 HU. The higher the threshold value was chosen, the smaller were the segmentation results. The deviation values varied more when the CT slices were thicker and when the threshold was more distant from the value of around -400 HU. However, a threshold that was too high had a stronger influence on the variation of the deviation values than a threshold that was too low.

The mean surface deviations between segmentation results and ground truth 3D-scan are shown in Fig. 7 together with the corresponding 6σ-intervals. Each line in the diagrams reflects one layer thickness of the CT DICOM data sets (0.4, 0.6, 1.5, 3.0, and 5.0 mm). The X-axis indicates the threshold chosen for segmentation in HU. Additionally, the kernel and the software used are differentiated. No values are plotted for segmentations that had a relative volume error exceeding -42.26%. A detailed analysis for the segmentations highlighted with numbers in Fig. 7 is provided in Figure 13 to Figure 16 of Appendix A, including surface deviation heat maps and histograms illustrating the relative frequency of deviation values. Figure 13 shows the influence of software and slice thickness for a soft tissue kernel and a given threshold of -600 HU. Figure 14 shows the influence of software and slice thickness for a bone kernel and a given threshold of -600 HU. Figure 15 shows the influence of software and kernel for a slice thickness of 0.4 mm and a given threshold of 0 HU. Figure 16 shows the influence of software and kernel for a slice thickness of 5.0 mm and a given threshold of 0 HU.

### Evaluation of the DEE

**Fig. 8:**
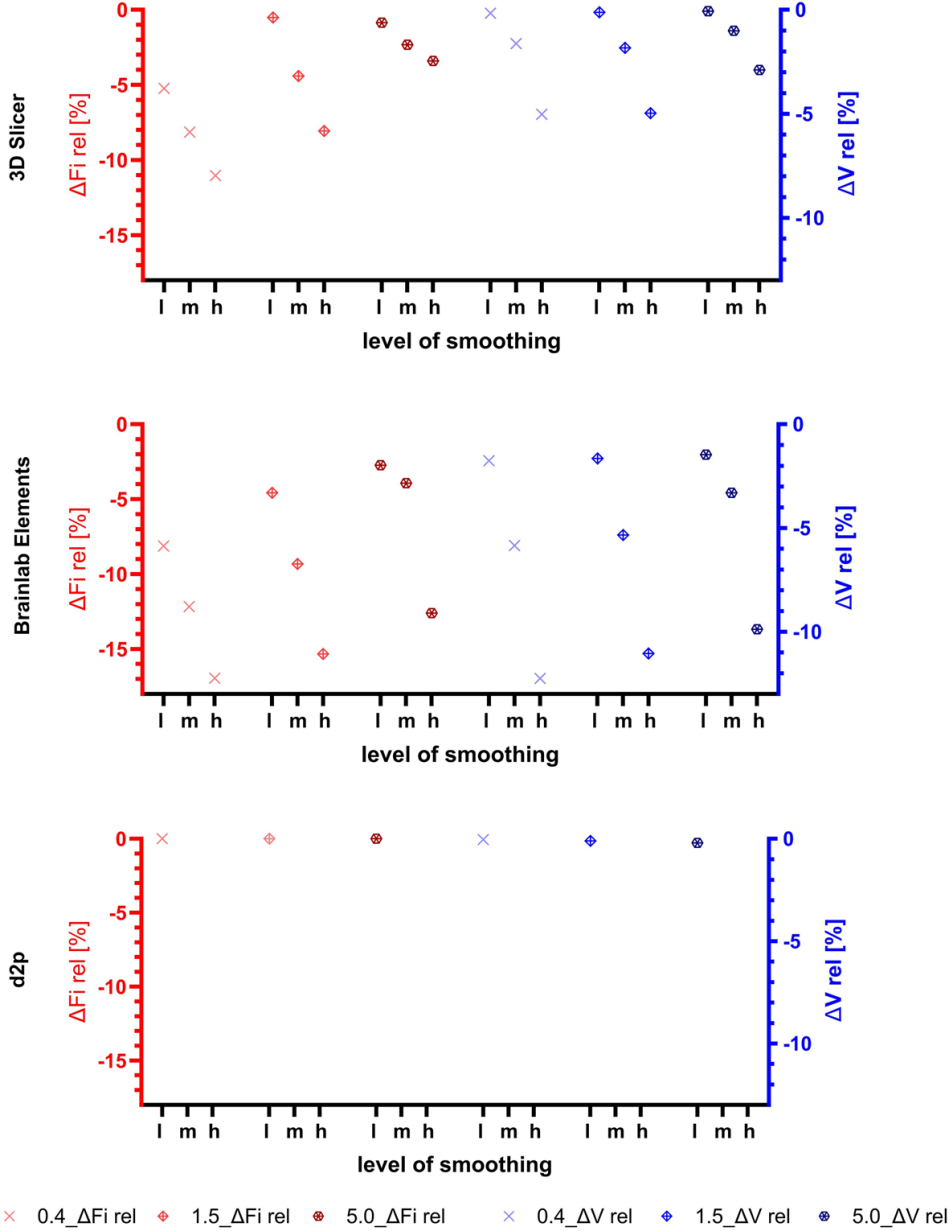
Relative change of file size (∆Fi rel) and volume (∆V rel) caused by smoothing of three different levels. l: low, m: medium, h: high. The symbols and their color indicate the slice thickness of the DICOM datasets underlying the segmentations (0.4, 1.5 and 5.0 mm), which were then smoothed to determine the DEE

**Fig. 9:**
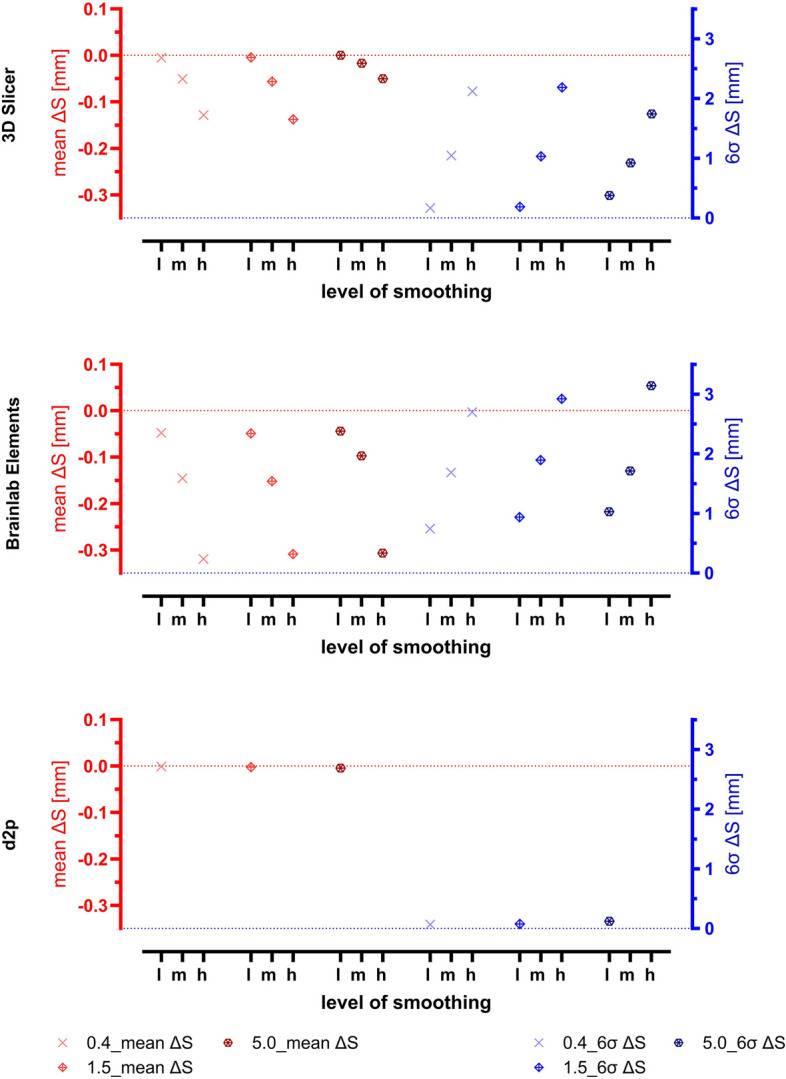
Means (mean ∆S) and 6σ-intervals (6σ ∆S) of the surface deviation between direct segmentation results and print-STLs caused by smoothing of three different levels. l: low, m: medium, h: high. The symbols and their color indicate the slice thickness of the DICOM datasets underlying the segmentations (0.4, 1.5 and 5.0 mm), which were then smoothed to determine the DEE. A more detailed analysis of the effects caused by smoothing is shown in Fig. 17

The relative change of file size (∆Fi rel) caused by the smoothing process is shown in Fig. 8 together with the relative change of volume (∆V rel). Negative values indicate a reduction of file size or volume. The mean surface deviations between direct segmentation results and print-STLs (results of the smoothing process) are shown in Fig. 9 together with the corresponding 6σ-intervals.

In summary, the relative reduction in STL file size was greater for higher levels of smoothing and thinner slice thickness of the underlying DICOM datasets. The same refers to the relative reduction in volume although the influence of slice thickness on the volume reduction was less than on the file size. In contrast, the slice thickness of the corresponding DICOM dataset did not significantly affect the surface deviation caused by the smoothing process in terms of means and variation (described by the 6σ-intervals). However, the level of smoothing had a significant effect, the model was shrinked with increasing level of smoothing. In addition, the 6σ-intervals increased with higher smoothing levels. Conversely, the strongest smoothing setting available in D2P had only a small effect on ∆Fi rel, ∆V rel, mean ∆S and 6σ ∆S.

A more detailed analysis of the effects caused by smoothing is shown in Figure 17 of Appendix A, including the effect of three different smoothing levels at a given slice thickness of 0.4 mm and the effects of software and slice thickness at a given smoothing level “medium”.

### Evaluation of the PrE

**Fig. 10:**
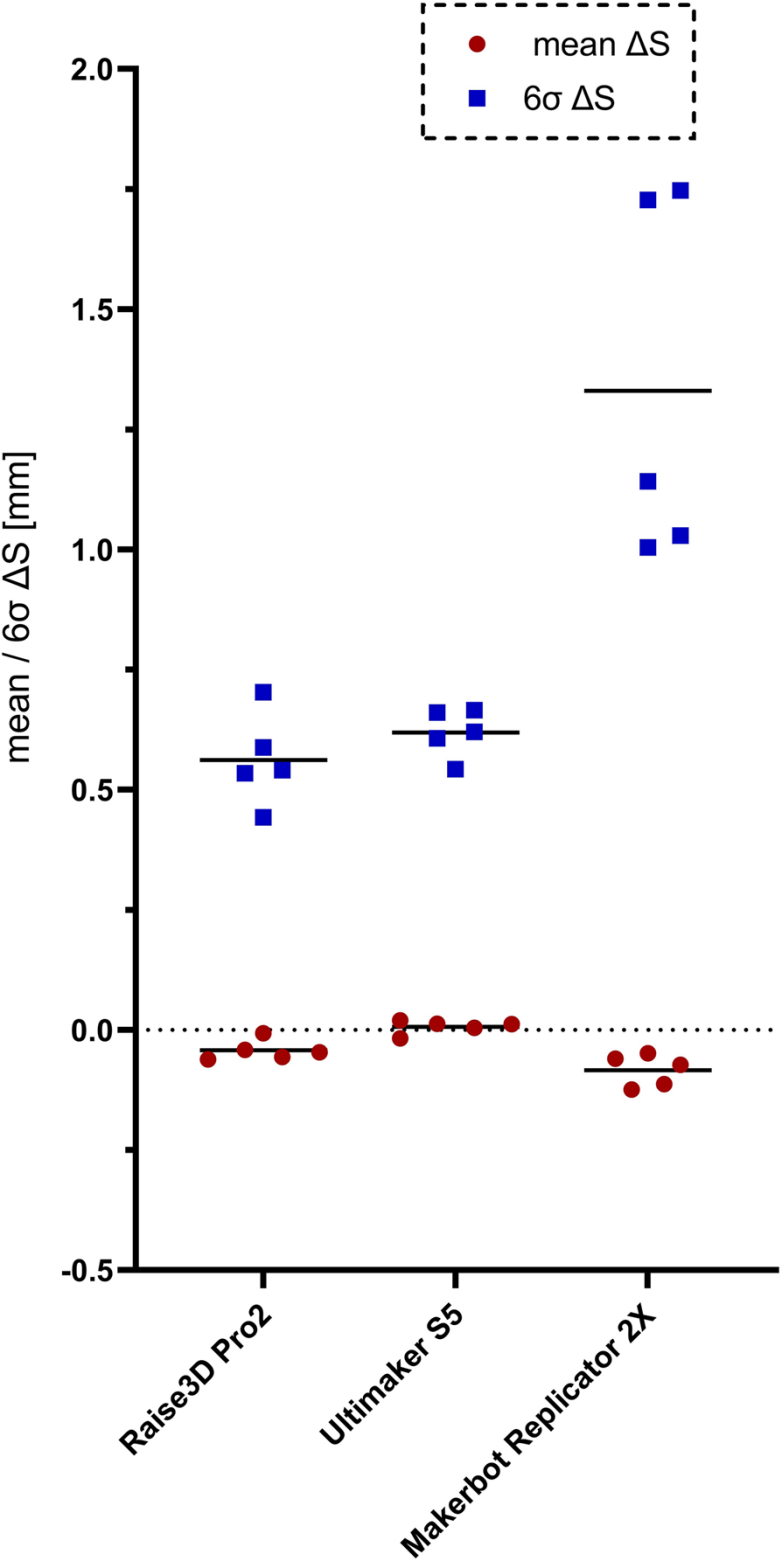
means (mean ∆S) and 6σ-intervals (6σ ∆S) of the surface deviation between construction STLs and 3D-scans of printed models. A detailed analysis of the PrE of the least accurate printer (Replicator 2X) is provided in Fig. 18 of Appendix A

The mean surface deviations between construction STLs and 3D-scans of the printed models (mean ∆S) is shown in Fig. 10 for each of the printers together with the corresponding 6σ-intervals (6σ ∆S).

In summary, the accuracy of the Ultimaker S5 and the RaisePro2 was of comparably high accuracy, whereas the Replicator 2X was notably inferior. The most accurate results were achieved with the Ultimaker S5. A detailed analysis of the PrE of the least accurate printer (Replicator 2X) is provided in Figure 18 of Appendix A.

### Evaluation of the total error

Parameter configuration 1 is presented as an optimal setup (referring to the results of this study) with a threshold of -400 HU, a slice thickness of 0.4 mm, and low-level smoothing. In this case, the mean surface deviation for the total error is 0.0093 mm with a standard deviation of 0.2265 mm. Accordingly, the 6σ ∆S is 1.3588 mm.

**Fig. 11:**
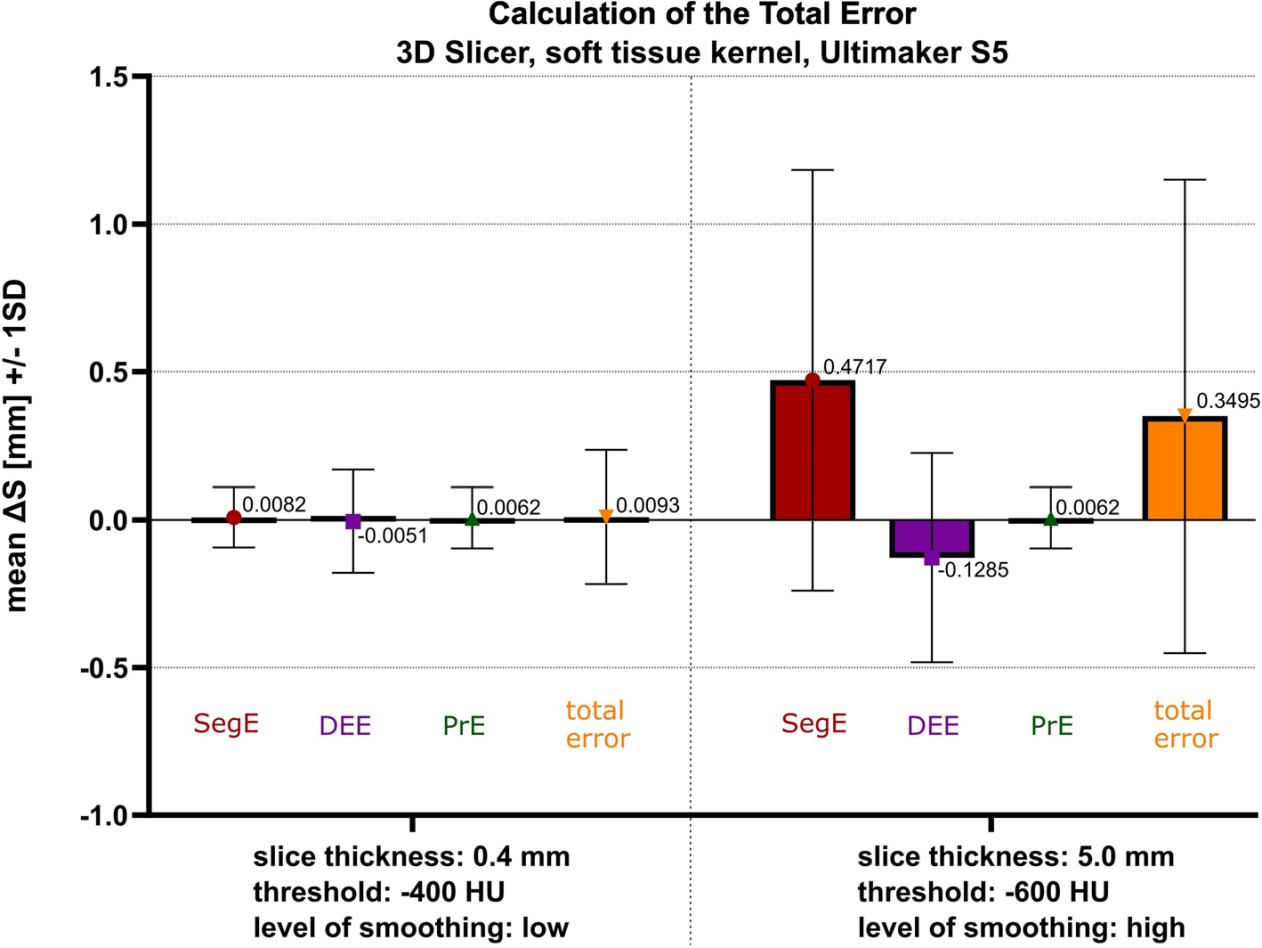
Total error for both parameter configurations, calculated as sum of partial errors

Parameter configuration 2 is presented as an example when only image data with a slice thickness of 5.0 mm is available, which would be considered insufficient as a data source for 3D-printing. A lower threshold of -600 HU is chosen together with a high-level of smoothing for compensation. In this case, the mean surface deviation for the total error is 0.3494 mm with a standard deviation of 0.8001 mm. Accordingly, the 6σ ∆S is 4.8055 mm. The partial errors and the calculated total errors are visualized for both parameter configurations in Fig. 11.

## Discussion

We observed a linear decrease in the relative volume error (∆V rel) in the range from -800 to 0 HU. Above the threshold of 0 HU, there was a considerably sharper decrease in ∆V rel. This is primarily due to the surface no longer being closed beyond a certain threshold (slightly above 0 HU in our study, scanning PLA in air). Although Friedli et al. investigated the effect of threshold values on the deviation of segmentations from a reference segmentation within a much narrower range of 80 HU and observed mean surface deviations of up to 0.95 mm, our findings (mean surface deviation values are presented in Fig. 7) are consistent with their work, as it is important to note that their evaluation is based on real patient data that includes adjacent soft tissue [26]. This results in a higher impact of threshold changes on the geometric deviation of the segmentation results. Another aspect they investigated is the comparison of the effects of threshold adjustments on computed tomography (CT) with cone beam computed tomography (CBCT) based segmentations. They found that CBCT based segmentations were slightly less sensitive to these changes. Methodologically, their approach differed in comparing segmentation results to a reference segmentation rather than to a ground truth 3D-scan, which presents both a limitation and an opportunity: A limitation of their approach is that it does not allow conclusions about the absolute deviation from the original structure. However, their method positions the reference segmentation and the test segmentations in the same coordinate system. This allows to infer from the transformation matrix of the iterative closest point alignment whether threshold settings affect the spatial position of the segmentation results.

Regarding the influence of threshold levels, our results further indicate that the optimal threshold is minimally affected by the slice thickness of the underlying CT scans (the graphs for mean ∆S and ∆V rel in Figs. 6 and 7 cross the zero line for all slice thicknesses within a very narrow threshold range). However, the curves become steeper as slice thickness increases. This suggests that segmentations based on thicker-slice CTs are more sensitive to threshold changes than those based on thin-slice CTs, resulting in segmentations from thicker-slice CTs reaching the volume cutoff at lower thresholds than segmentations from thin-slice CTs. Furthermore, it is evident that segmentations based on BK CTs are notably more sensitive to threshold changes above 0 HU compared to STK based segmentations. This can be attributed to the inferior signal- and contrast-to-noise ratio of BK CTs [21]. In general, the segmentation result becomes larger than the reference at too low thresholds, and smaller at too high thresholds.

The file size remains constant across a wide range of threshold values for all software and kernels, but there is a noticeable difference between the CT slice thicknesses. Ranking the CT slice thicknesses by the file size of the segmentation results, from smallest to largest, the order is as follows 5.0 mm, 1.5 mm, 0.6 mm, 3.0 mm, 0.4 mm. In general, it can be seen that higher resolution in CT means more polygons in the segmentation result. However, the 3 mm slice thickness seems to involve a specific staircase effect, resulting in its segmentation results containing more polygons than those based on 0.6 and 1.5 mm slice thicknesses. When the surface is no longer completely closed, certain effects appear (e.g., a particularly large increase in file size from threshold 0 to threshold 100). This is especially true for segmentations based on BK CTs. Here, we observe not only a particularly strong increase in file size above 0 HU but also a change in the order of CT slice thicknesses when ranked by the file size of the corresponding segmentations (5.0, 3.0, 1.5, 0.6, 0.4 mm). Additionally, it is noticeable that after the peak in file size at 0 HU, a decrease is observed. Both effects can be attributed to "island formation" and the non-enclosed surface at too high thresholds. Due to the higher noise in BK CTs, the effect of island formation is particularly strong and significantly affects the number of polygons in the segmentation results. However, as the threshold is further increased, the number of individual islands that are part of the segmentation decreases, which in turn reduces the number of polygons.

Mean ∆S and 6σ ∆S of the SegE (representing deviations between direct segmentation result and reference 3D-scan) are shown in Fig. 7. Fundamentally, we observe similar effects for mean ∆S as with ∆V rel, though this parameter, especially in combination with 6σ ∆S, provides more detailed information about the surface deviation.

Our results suggest that when a threshold is set too low, the deviation of the segmentation result from the reference becomes more positive as the slice thickness increases. This finding also allows further conclusions to be drawn from the results of other authors: Eliyahu et al. investigated how the slice thickness in CT affects the results of CT-based finite element analysis to assess the load capacity of the femur [27]. In this context, they also investigated the extent to which the volume of the segmentation result depends on the slice thickness in underlying CT data. They observed that segmentations based on a 3 mm slice thickness have a larger volume than segmentations based on a 1 mm slice. This indicates that even with optimal slice thickness, the segmentation parameters employed in their study may slightly overestimate the true volume.

Huang et al. also investigated the influence of slice thickness on segmentation results [28]. In their study, CT data sets of the neck and head of 11 patients were reconstructed with various parameters, including 14 different slice thicknesses ranging from 0.6 mm to 10 mm, and were then segmented. To evaluate the deviation between segmentations based on various slice thicknesses to a reference segmentation based on a slice thickness of 3 mm, they utilized the parameters mean surface distance, Hausdorff distance, and Dice similarity coefficient. As expected, the difference in mean surface distance between the reference segmentation and the test segmentations increased as the slice thickness deviated from the reference. However, the Dice similarity coefficient and Hausdorff distance parameters were far more sensitive to changes in the slice thickness of the underlying DICOM datasets. Together with our results, this emphasizes that the mean surface distance alone is not sufficient to assess the deviation between two objects. This is particularly evident in Fig. 7, around a threshold of 400 HU, where the mean ∆S for all slice thicknesses are close together, yet the variability of deviation values, expressed through the parameter 6σ ∆S, differs greatly. We observe that the variability increases with thicker CT slice thicknesses, which can be attributed to staircase effect in the segmentation results. This is detailed in Figure 13 and Figure 14 from Appendix A, where another aspect becomes apparent: Despite exclusively using simple threshold segmentations across all software variants, segmentations based on a 5 mm slice thickness in Brainlab and D2P exhibit less staircase formation compared to those in 3D Slicer. The lower values for 6σ ∆S of the Brainlab Elements and D2P segmentations compared to the 3D Slicer segmentations in Fig. 7 also reflect this. Therefore, it can be concluded that D2P and Brainlab, even when performing simple threshold segmentation, include algorithms that optimize segmentation results based on DICOM data with thicker slices. Akmal et al. observed a similar phenomena: In their study on cumulative errors in the process for producing patient-specific implants, they found that staircase formation in the segmentation result correlates most closely with the standard deviation of the surface deviation [29].

Overall, despite minor differences in surface structure among segmentations performed with different software, no single software was found to be notably superior or inferior when parameters were comparably chosen. This aligns with the findings of Kamio et al. [15], who compared nine software variants for mandibular segmentation through surface comparison and found average deviation values of less than 0.5 mm, with maximum values ranging between -1.55 and 1.75 mm. Moreover, no statistically significant differences were found between the software options.

Around the optimal threshold, no noticeable differences between kernels were observed across all software, aligning with the findings of Ogden et al. [30]. They conducted a CT-scan of a 3D-printed cube and reconstructed it using different kernels, finding a maximum deviation between DICOM data of 1.18 mm. However, their study focused on linear measurements in DICOM data and did not directly investigate the impact on segmentation. Nonetheless, the influence of threshold levels on the segmentation of a L1 lumbar vertebra scanned in air was examined for thresholds of 125, 150, 175, and 200 HU, where average deviations were found to be in the sub-millimeter range.

In our study, another effect was observed for segmentations based on BK CT data: Although the graphs of mean ∆S are generally steeper for thicker slice thicknesses, the mean surface deviations of BK-based segmentations increase stronger for thin slice thicknesses above a threshold of 0 HU. This observation can be attributed to the poorer contrast-to-noise ratio in BK: As seen in Fig. 6, BK segmentations from a threshold of 0 HU onwards exhibit a more complex surface than soft tissue kernel (STK) segmentations, which can be traced back to island formation. In the surface comparisons, the deviation was determined by searching for the corresponding point on the 3D-scan from every point on the surface of the segmentation result. Since BK has significantly more "internal islands" (with negative deviation from the reference surface) than STK, mean ∆S for BK becomes markedly more negative than for STK. The same applies to the dispersion of the deviation values, which is highest for slice thicknesses of 0.4, 0.6 around the threshold of 0 HU (or beyond, which could not be reliably measured). This effect is further illustrated in Figure 15 from Appendix A, where a direct comparison between STK and BK for a threshold of 0 HU and a slice thickness of 0.4 mm is shown. It is evident that with STK the surface integrity is largely preserved and deviation values are normally distributed. In contrast, with BK, a large portion of the surface is slightly more accurate, but this is contrasted by considerable indentations with negative deviations and internal island formations, resulting in deviation values that are no longer normally distributed. Akmal et al. reached a similar conclusion in their study: They preferred a softer kernel (J30S) over a harder one (J70H) for threshold-based segmentation of a CT-scanned dry skull, as the harder kernel produced more artefacts [29]. The recommendations of Materialise not to perform segmentations of bony structures on DICOM data based on hard kernels are consistent with their results [31]. Dzierżak et al. provide further evidence supporting this aspect: They explored how the reconstruction kernel of CT data affects the accuracy of an automatic model for detecting osteoporosis in CT scans of the lumbar to sacral spine [32]. For the analysis of the image data, they used the software MaZda and concluded that the accuracy of osteoporosis detection is better with a soft kernel than with a hard kernel. They attribute this to the focus on analyzing the trabecular bone, which is atypically demineralized in the context of osteoporosis, and thus is more accurately represented by a soft tissue kernel. This is further supported by Giambini et al. [33]. In the context of quantitative computed tomography, according to their findings, soft kernels are more suitable than hard kernels for estimating bone mineralization. Although soft kernels may slightly round edges and corners, they have less noise, which is also of great relevance in the context of segmentation. Jiang et al., on the other hand, see that both bone kernel and soft tissue kernel are suitable for the CT-based finite element method for screening of osteoporosis [34]. However, they observe kernel-specific threshold values and recommend standard (soft tissue) kernels due to their wider use.

Another aspect that should be considered in terms of segmentation accuracy is the partial volume effect (PVE). The extent of its impact depends, among other factors, on contrast and slice thickness. Neubauer et al [35] investigated this for kidney stones and concluded that algorithms to compensate for PVE do not significantly improve the accuracy of segmentation results.

Regarding the relative change in file size (∆Fi rel) induced by smoothing with different smoothing factors, and for segmentations based on CTs with varying slice thicknesses, the relative change in file size was greater the thinner the CT slices and the higher the smoothing factor (Fig. 8).

The primary influence on the relative change in volume was the smoothing factor. Generally, the relative volume changes caused by smoothing were negative, indicating that smoothing tends to reduce the size of the models (Fig. 8). In terms of mean ∆S and 6σ ∆S, these were quite stable in relation to the slice thickness of the underlying CTs, but significantly dependent on the smoothing factor: The greater the smoothing factor, the more negative the mean surface deviation and the greater the variance. However, for D2P, there is a peculiar case where even the strongest possible smoothing factor had almost no effect (Fig. 9). Figure 17 from Appendix A further illustrates, how smoothing affects surface deviation: It mainly affects edges and corners, with larger surfaces being less affected.

Fogarasi et al. found no statistical differences between the final models of mandibular tumor segmentations that were smoothed with four different software solutions [36]. Their analysis was based on the average of three smoothing levels within each software and they employed "union and intersection figures" to analyze similarities and differences between the software. In addition to the fact that the impact of smoothing in the context of the medical 3D-printing process has not been well studied [13], another challenge becomes apparent: There is no uniform scale for the smoothing factor and no standardized naming of the parameters that define the extent of smoothing. As a result, it is virtually impossible to compare the absolute deviation values caused by smoothing between different software solutions. In fact, it is questionable whether different versions of the same software can be directly compared, since it is unclear to the operator how the scaling of the parameter affecting the degree of smoothing may have changed. More uniform standards would be desirable here.

Regarding the PrE it became evident that among the three FFF printers, the adhesion of the printed model to the build plate had the most significant impact on the printing accuracy. The Ultimaker S5 utilized a heated glass build plate (60°C) with an additional adhesive spray, the Raise3D Pro2 employed a heated metal build plate (60°C), and the Replicator 2X's build plate could only be heated at the beginning of the printing process. While all three printers produced precise models, the results from the Replicator 2X were notably affected by warping. Ramian et al. in their study on "Thermal Deformations of Thermoplast during 3D-Printing" demonstrated that the effect of warping is highly dependent on the temperature of the print bed [37]: They conducted test prints and gradually increased the temperature of the print bed in 5 °C steps from 75 ° to 100 °, observing average lift off values from 0.89 to 1.51 mm. However, they printed with Acrylonitrile Butadiene Styrene Copolymer (ABS), which is even more susceptible to warping compared to PLA. Figure 18 in Appendix A provides a more detailed illustration of the warping that has occurred with the Replicator 2X. This allows for further insights into why two models exhibited significantly more warping than the remaining three (with 6σ ∆S around 1.1 mm and around 1.7 mm). The two models that were positioned on the left and right outer edges of the print bed experienced particularly strong vertical lift-off. This suggests that the heat distribution in the print bed was uneven, and the outer areas may have cooled down more quickly.

Generally, our results for the PrE were within the range described in the literature. In a recently published review by Schulze et al. [13] with 139 publications included, the median printing error was 0.26 mm, and the results, which were based on a surface deviation analysis, ranged between 0.025 and 0.5 mm, compared to 0.12 mm as the maximal absolute mean deviation value of this study.

In this publication, the numerous parameters that affect the PrE were not examined in detail; however, our data clearly illustrate how significant the effects of individual parameter changes can be in terms of PrE. For FFF 3D-printing, Brion et al. propose an innovative approach to deal with this [38]: They acquired detailed video recordings, including thermal images, of FFF printing processes. These were labeled with the corresponding printing parameters and used to train a neural network that enables real-time detection of printing errors and effective automatic optimization of printing parameters for various printers, materials, and geometries.

Akmal et al. presented a similar approach to calculate the total error according to rules of error propagation [29]. In contrast to our approach, they did not consider the effect of the DEE (in their study: 3D modelling error) separately for the calculation of the total error.

**Fig. 12:**
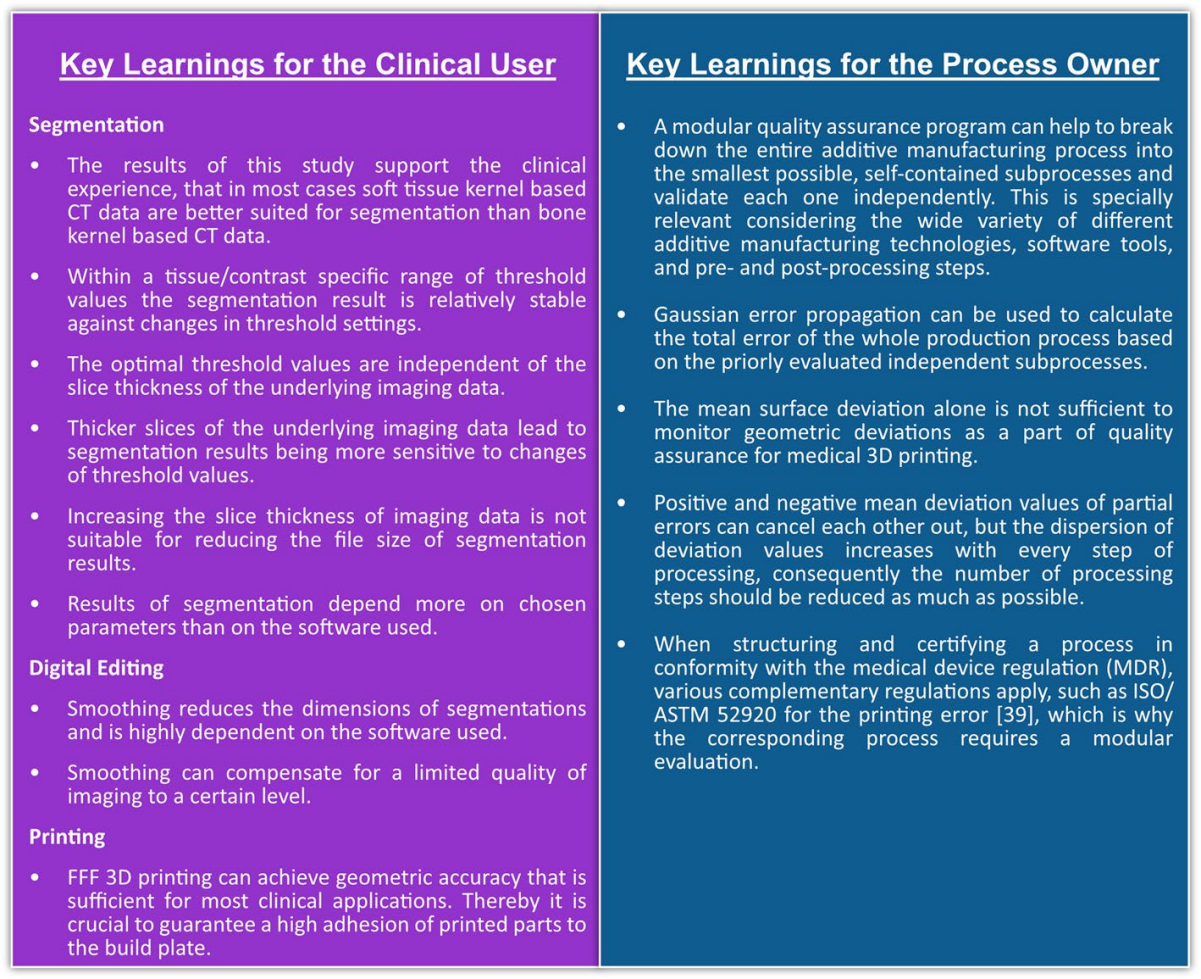
Key learnings for the clinical user and the process owner in medical 3D printing at the point of care [39]

**Fig. 13:**
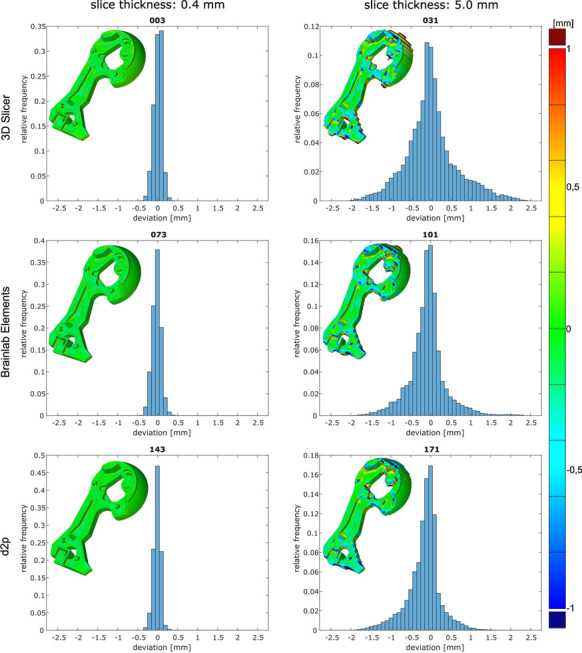
Influence of software and slice thickness for a soft tissue kernel and a given threshold of -600 HU. The segmentation results are shown in the upper left corner of each diagram, and the heat maps indicate the deviation from the ground truth 3D-scan according to the legend on the right

**Fig. 14:**
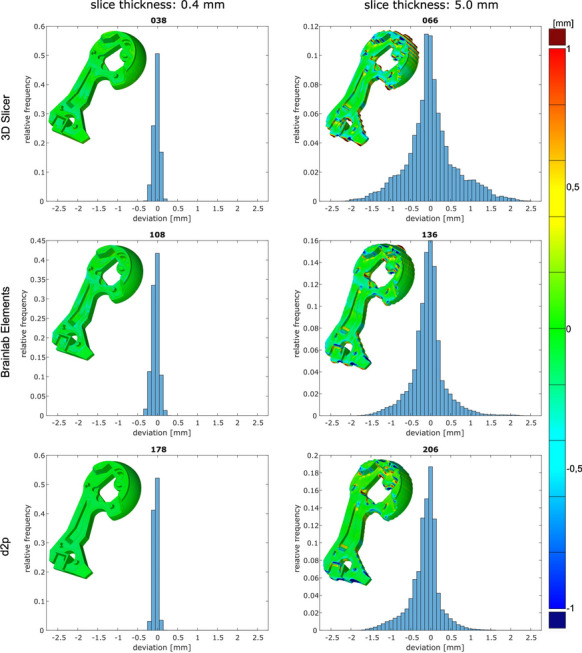
Influence of software and slice thickness for a bone kernel and a given threshold of -600 HU. The segmentation results are shown in the upper left corner of each diagram, and the heat maps indicate the deviation from the ground truth 3D-scan according to the legend on the right

**Fig. 15:**
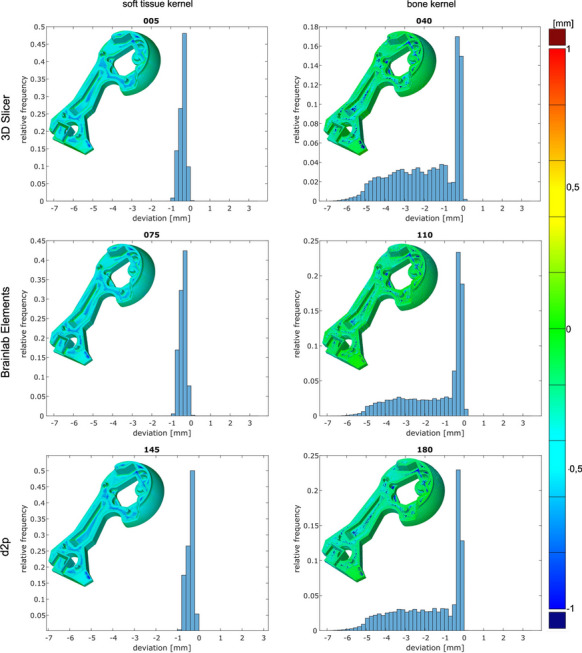
Influence of software and kernel for a slice thickness of 0.4 mm and a given threshold of 0 HU. The segmentation results are shown in the upper left corner of each diagram, and the heat maps indicate the deviation from the ground truth 3D-scan according to the legend on the right

**Fig. 16:**
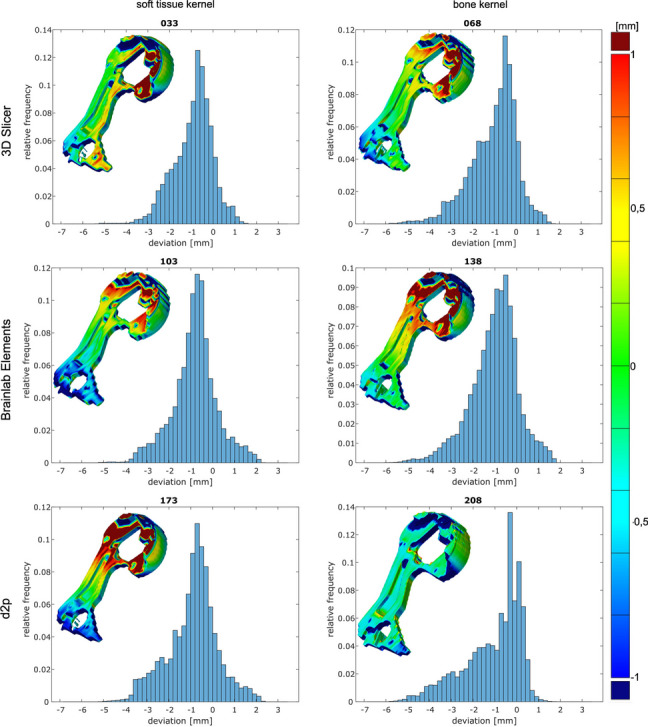
Influence of software and kernel for a slice thickness of 5.0 mm and a given threshold of 0 HU. The segmentation results are shown in the upper left corner of each diagram, and the heat maps indicate the deviation from the ground truth 3D-scan according to the legend on the right

**Fig. 17:**
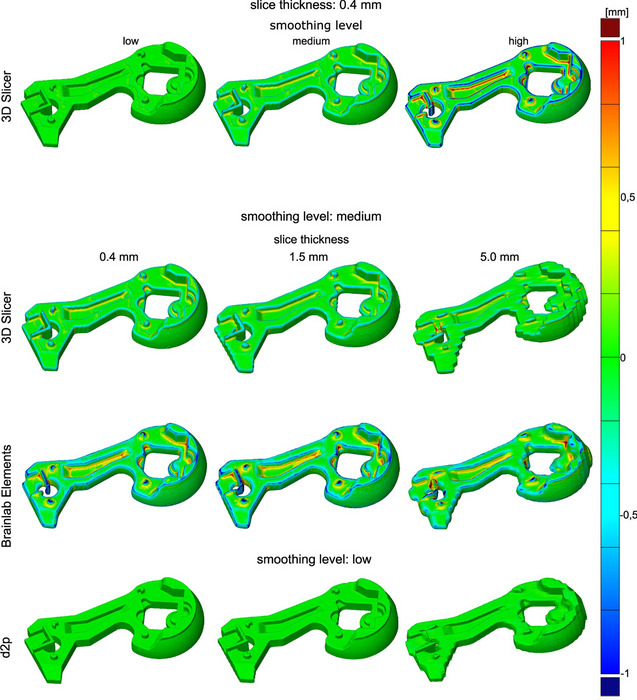
Effect of three different smoothing levels and slice thickness on the DEE. The heat maps indicate the deviation be-tween direct segmentation results and print-STLs according to the legend on the right. first row: effect of different smoothing levels for a given slice thickness of the underlying DICOM data set and software 3D Slicer. Second and third row: effect of different slice thicknesses of the underlying DICOM data set on the DEE for a given smoothing level (medium). Note that in D2P only a low level of smoothing with a minimal effect is available

**Fig. 18:**
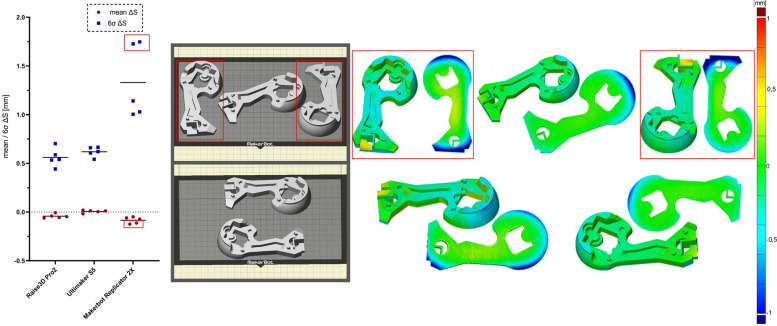
PrE analysis of the Replicator 2X prints. Two outliers are highlighted with red boxes. The heat maps indicate the deviation between construction STLs and 3D-scans of printed models according to the legend on the right. The heat maps are arranged according to the print bed orientation of the corresponding printed models. For each of the five printed models, a view from above and a view from below are presented. In particular, the heat maps corre-sponding to the two outliers from the plots in Figure 10 clearly show that more severe warping has occurred

Other differences to our approach include their use of the head of *Sus domesticus* as the original structure for imaging (including soft tissue) and their calculation of the total error for an implant. Since this implant has two boundary surfaces, they doubled the thresholding error and then reported a worst-case total error for the implant of 2.31 mm with a standard deviation of 0.79 mm (compared to our results of 0.01 ± 0.23 mm and 0.35 ± 0.80 mm, respectively). Considering their value for a single boundary surface (1.71 mm), both their results and ours fall within the range reported by Schulze et al. for the total error (0.05-2.25 mm) [13]. Figure 12 summarizes the key learnings for the clinical user and the process owner based on the results of this study.

### Limitations

The main limitation of our study lies in the imaging setup. According to the categorization by Schulze et al., scanning artificial models in air is the least realistic imaging setup for assessing the SegE [13]. Although the direction and relative strength of the observed effects are generalizable, the absolute deviation values of the SegE in this study only refer to the contrast of PLA to air. Yet in this study, the highly controlled experimental setup also provides an opportunity to evaluate the effect of different parameters on segmentation accuracy in the absence of confounding factors. However, due to the limited realism of our approach, further research is needed to transfer our results to more realistic imaging setups. Synthetic DICOM datasets or simulation of soft tissue may be used for this purpose [40, 41]. For the modality CT, tube voltage and tube current may be taken into account in future research. These were not investigated in this study. Our study focused specifically on CT data as the most common source for medical 3D-printing [14]. Similar research regarding the influence of other modalities, such as MRI or 3D ultrasound, on the 3D-printing process is needed. Additionally, future research could consider a broader range of software including Mimics (Materialise, Belgium). Also, our study focused only on FFF-3D-printing. Therefore, our results could be complemented with research on further printing technologies such as vat photopolymerization and material jetting. Due to the shape of the benchy, no support was required during the printing process, which prevented confounding by the slicing software However, the removal of support structures in the post-processing step represents a relevant, printing technology-dependent source of error that is not addressed in this publication.

## Conclusions

The three partial errors of the medical 3D-printing process segmentation error (SegE), digital editing error (DEE) and printing error (PrE) were evaluated separately. Specifically, for SegE, a variety of parameter configurations were explored, and the influence of threshold and slice thickness was analyzed across a wide range. Due to their relevance for many semi-automatic segmentation algorithms, valuable insights can be gained on how to adjust segmentation or smoothing parameters to minimize the total error even under suboptimal imaging conditions.

For the experimental setup with high contrast between the scanned object and its environment, soft tissue kernel-based segmentations are at least as accurate as bone kernel-based segmentations over a wide threshold range. However, since the latter tend to produce notably more artefacts at too high thresholds, soft kernels seem to be advantageous for segmentation in most cases. This is particularly true considering that segmentation on real image data requires higher threshold values due to the increased noise with hard kernels.

The printing error is strongly influenced by the adhesion of the printed model to the build plate, which can be greatly improved by optimal conditioning, e.g. heating the build plate with constant heat distribution.

The concept of Gaussian error propagation can increase the understanding of various influences on geometric deviations in medical 3D-printing. Negative and positive average values of the partial errors can cancel each other out, but the variation of the error values increases with each processing step. Future research, using more realistic imaging setups, can contribute to represent the total error as a function of the partial errors and their influencing parameters. Consequently, analytical approaches are conceivable to optimize downstream parameters depending on the characteristics of the imaging available. Similarly, they can be used to calculate the achievable quality of the results, which can help to optimize the imaging, so that a result sufficient for 3D-printing can be achieved with minimal radiation exposure.

## Supplementary Information

Supplementary Material 1.

## Data Availability

The data presented in this study are available upon request from the corresponding author. The data are not publicly available due to privacy restrictions.

## Appendix A

**Table 1:** Parameters of image acquisition

slice thickness (mm)	kilo voltage peak (kV)	tube current (mAs)	pixel spacing (mm)	kernel
0.4	120	92	0.31, 0.31	U30u
	120	92	0.31, 0.31	U70u
0.6	120	92	0.31, 0.31	U30u
	120	92	0.31, 0.31	U70u
1.5	80	56	0.32, 0.32	I26f
	80	56	0.32, 0.32	I70f
3.0	80	52	0.98, 0.98	B40f
	80	52	0.98, 0.98	B60f
5.0	80	56	0.32, 0.32	I30f
	80	56	0.32, 0.32	I70f

## Abbreviations

PSI: Patient specific instrument
DICOM: Digital imaging and communications in medicine
STL: Standard tessellation language
SegE: Segmentation error
DEE: Digital editing error
PrE: Printing error
IAE: Image acquisition error
SegC: Segmentation comparison error
FFF: Fused filament fabrication
CAD: Computer aided design
CT: Computed tomography
STK: Soft tissue kernel
BK: Bone kernel
ASCII: American Standard Code for Information Interchange
Fi: File size
l: Low
m: Medium
h: High
CBCT: Cone beam computed tomography
ABS: Acrylonitrile Butadiene Styrene Copolymer

## References

[1] Schulze M, Gosheger D, Bockholt S, de Vaal M, Budny T, Tönnemann M, Pützler J, Bövingloh AS, Rischen R, Hofbauer V, Lübben T, Deventer N, Ahrens H. Complex bone tumors of the trunk-the role of 3D printing and navigation in tumor orthopedics: a case series and review of the literature. J Pers Med. 2021;11:517. 10.3390/jpm11060517. 34200075 10.3390/jpm11060517 PMC8228871

[2] Valls-Esteve A, Tejo-Otero A, Lustig-Gainza P, Buj-Corral I, Fenollosa-Artés F, Rubio-Palau J, Barber-Martinez de La Torre I, Munuera J, Fondevila C, Krauel L. Patient-specific 3D printed soft models for liver surgical planning and hands-on training. Gels. 2023;9:339. 10.3390/gels9040339. 37102951 10.3390/gels9040339 PMC10138006

[3] Wong A, Goonewardene MS, Allan BP, Mian AS, Rea A. Accuracy of maxillary repositioning surgery using CAD/CAM customized surgical guides and fixation plates. Int J Oral Maxillofac Surg. 2021;50:494–500. 10.1016/j.ijom.2020.08.009. 32919821 10.1016/j.ijom.2020.08.009

[4] Yang C, Zhang C, Wu J, Xu X, Zhang Y, Zhang S. Three-dimensional printed customized surgical guides for the precise correction of complex midfacial post-traumatic deformities. J Craniofac Surg. 2022;33:1150–3. 10.1097/SCS.0000000000008329. 36041109 10.1097/SCS.0000000000008329

[5] Omar M, Schulze M, Bruns N, Kotrych D, Gosheger G, Ettinger M. Update 3D-Druck in der Chirurgie muskuloskeletaler Tumoren. Unfallchirurg. 2022;125:361–70. 10.1007/s00113-022-01160-5. 35312794 10.1007/s00113-022-01160-5

[6] Kotrych D, Angelini A, Bohatyrewicz A, Ruggieri P. 3D printing for patient-specific implants in musculoskeletal oncology. EFORT Open Rev. 2023;8:331–9. 10.1530/EOR-23-0066. 37158428 10.1530/EOR-23-0066 PMC10233802

[7] Grab M, Hundertmark F, Thierfelder N, Fairchild M, Mela P, Hagl C, Grefen L. New perspectives in patient education for cardiac surgery using 3D-printing and virtual reality. Front Cardiovasc Med. 2023;10:1092007. 10.3389/fcvm.2023.1092007. 36937915 10.3389/fcvm.2023.1092007 PMC10020687

[8] Joseph FJ, Vanluchene HER, Goldberg J, Bervini D. 3D-Printed Head Model in Patient’s Education for Micro-Neurosurgical Aneurysm Clipping Procedures. World Neurosurg. 2023;175:e1069–74. 10.1016/j.wneu.2023.04.070. 37087042 10.1016/j.wneu.2023.04.070

[9] Molinari G, Emiliani N, Cercenelli L, Bortolani B, Gironi C, Fernandez IJ, Presutti L, Marcelli E. Assessment of a novel patient-specific 3D printed multi-material simulator for endoscopic sinus surgery. Front Bioeng Biotechnol. 2022;10: 974021. 10.3389/fbioe.2022.974021. 36466346 10.3389/fbioe.2022.974021 PMC9712453

[10] Chedid VG, Kamath AA, Knudsen JM, Frimannsdottir K, Yost KJ, Geske JR, Morris JM, Taner T, Matsumoto JM, Kamath PS. Three-dimensional-printed liver model helps learners identify hepatic subsegments: a randomized-controlled cross-over trial. Am J Gastroenterol. 2020;115:1906–10. 10.14309/ajg.0000000000000958. 33156110 10.14309/ajg.0000000000000958

[11] Yammine K, Karbala J, Maalouf A, Daher J, Assi C. Clinical outcomes of the use of 3D printing models in fracture management: a meta-analysis of randomized studies. Eur J Trauma Emerg Surg. 2022;48:3479–91. 10.1007/s00068-021-01758-1. 34383092 10.1007/s00068-021-01758-1

[12] Meyer-Szary J, Luis MS, Mikulski S, Patel A, Schulz F, Tretiakow D, Fercho J, Jaguszewska K, Frankiewicz M, Pawłowska E, Targoński R, Szarpak Ł, Dądela K, Sabiniewicz R, Kwiatkowska J. The role of 3D printing in planning complex medical procedures and training of medical professionals-cross-sectional multispecialty review. Int J Environ Res Public Health. 2022;19:3331. 10.3390/ijerph19063331. 35329016 10.3390/ijerph19063331 PMC8953417

[13] Schulze M, Juergensen L, Rischen R, Toennemann M, Reischle G, Puetzler J, Gosheger G, Hasselmann J. Quality assurance of 3D-printed patient specific anatomical models: a systematic review 3D. 3D Print Med. 2024;10:9. 10.1186/s41205-024-00210-5. 38536566 10.1186/s41205-024-00210-5 PMC10967057

[14] Chepelev L, Wake N, Ryan J, Althobaity W, Gupta A, Arribas E, Santiago L, Ballard DH, Wang KC, Weadock W, Ionita CN, Mitsouras D, Morris J, Matsumoto J, Christensen A, Liacouras P, Rybicki FJ, Sheikh A. Radiological Society of North America (RSNA) 3D printing Special Interest Group (SIG): guidelines for medical 3D printing and appropriateness for clinical scenarios. 3D Print Med. 2018;4:11. 10.1186/s41205-018-0030-y. 30649688 10.1186/s41205-018-0030-y PMC6251945

[15] Kamio T, Suzuki M, Asaumi R, Kawai T. DICOM segmentation and STL creation for 3D printing: a process and software package comparison for osseous anatomy, 3D Print. Med. 2020;6:17. 10.1186/s41205-020-00069-2. 10.1186/s41205-020-00069-2 PMC7393875 32737703

[16] Joskowicz L, Cohen D, Caplan N, Sosna J. Inter-observer variability of manual contour delineation of structures in CT. Eur Radiol. 2019;29:1391–9. 10.1007/s00330-018-5695-5. 30194472 10.1007/s00330-018-5695-5

[17] Heye T, Merkle EM, Reiner CS, Davenport MS, Horvath JJ, Feuerlein S, Breault SR, Gall P, Bashir MR, Dale BM, Kiraly AP, Boll DT. Reproducibility of dynamic contrast-enhanced MR imaging. Part II. Comparison of intra- and interobserver variability with manual region of interest placement versus semiautomatic lesion segmentation and histogram analysis. Radiology. 2013;266:812–21. 10.1148/radiol.12120255. 23220891 10.1148/radiol.12120255

[18] Galvez M, Montoya CE, Fuentes J, Rojas GM, Asahi T, Currie W, Kuflik M, Chahin A. Error measurement between anatomical porcine spine, CT images, and 3D printing. Acad Radiol. 2020;27:651–60. 10.1016/j.acra.2019.06.016. 31326309 10.1016/j.acra.2019.06.016

[19] Nguyen P, Stanislaus I, McGahon C, Pattabathula K, Bryant S, Pinto N, Jenkins J, Meinert C. Quality assurance in 3D-printing: a dimensional accuracy study of patient-specific 3D-printed vascular anatomical models. Front Med Technol. 2023;5:1097850. 10.3389/fmedt.2023.1097850. 36824261 10.3389/fmedt.2023.1097850 PMC9941637

[20] Veiga-Canuto D, Cerdà-Alberich L, Sangüesa Nebot C, Martínez de Las Heras B, Pötschger U, Gabelloni M, Carot Sierra JM, Taschner-Mandl S, Düster V, Cañete A, Ladenstein R, Neri E, Martí-Bonmatí L. Comparative multicentric evaluation of inter-observer variability in manual and automatic segmentation of neuroblastic tumors in magnetic resonance images. Cancers. 2022;14:3648. 10.3390/cancers14153648. 35954314 10.3390/cancers14153648 PMC9367307

[21] Paul J, Krauss B, Banckwitz R, Maentele W, Bauer RW, Vogl TJ. Relationships of clinical protocols and reconstruction kernels with image quality and radiation dose in a 128-slice CT scanner: study with an anthropomorphic and water phantom. Eur J Radiol. 2012;81:e699-703. 10.1016/j.ejrad.2011.01.078. 21316888 10.1016/j.ejrad.2011.01.078

[22] Lasek J, Piórkowski A. CT scan transformation from a sharp to a soft reconstruction kernel using filtering techniques. In: Singh SK, Roy P, Raman B, Nagabhushan P, editors. Computer Vision and Image Processing. Singapore: Springer Singapore; 2021. p. 56–65.

[23] Meng D, Wang Z, Bai C, Ye Z, Gao Z. Assessing the effect of scanning parameter on the size and density of pulmonary nodules: a phantom study. BMC Med Imaging. 2024;24:12. 10.1186/s12880-023-01190-4. 38182987 10.1186/s12880-023-01190-4 PMC10768218

[24] Schulze M, Gosheger G, Bockholt S, de Vaal M, Budny T, Tönnemann M, Pützler J, Bövingloh AS, Rischen R, Hofbauer V, Lübben T, Deventer N, Ahrens H. Complex bone tumors of the trunk—the role of 3d printing and navigation in tumor orthopedics: A case series and review of the literature. J Pers Med. 2021;11:517. 10.3390/jpm11060517. 34200075 10.3390/jpm11060517 PMC8228871

[25] Papula L. Fehlerfortpflanzung“ nach Gauß. In: Papula L, (Ed.). Mathematik für Ingenieure und Naturwissenschaftler Band 3 Vektoranalysis, Wahrscheinlichkeitsrechnung, mathematische Statistik, Fehler- und Ausgleichsrechnung, 3., verb. Aufl. Braunschweig: Vieweg; 1999. p. 674–689. 10.1007/978-3-322-94316-3.

[26] Friedli L, Kloukos D, Kanavakis G, Halazonetis D, Gkantidis N. The effect of threshold level on bone segmentation of cranial base structures from CT and CBCT images. Sci Rep. 2020;10:7361. 10.1038/s41598-020-64383-9. 32355261 10.1038/s41598-020-64383-9 PMC7193643

[27] Eliyahu L, Yosibash Z, Avivi I, Cohen YC, Ariel G, Sadovnic O, Sternheim A. On the influence of computed tomography’s slice thickness on computer tomography based finite element analyses results. Clin Biomech (Bristol, Avon). 2023;102:105889.10.1016/j.clinbiomech.2023.105889 36774735 10.1016/j.clinbiomech.2023.105889

[28] Huang K, Rhee DJ, Ger R, Layman R, Yang J, Cardenas CE, Court LE. Impact of slice thickness, pixel size, and CT dose on the performance of automatic contouring algorithms. J Appl Clin Med Phys. 2021;22:168–74. 10.1002/acm2.13207. 33779037 10.1002/acm2.13207 PMC8130223

[29] Akmal JS, Salmi M, Hemming B, Teir L, Suomalainen A, Kortesniemi M, Partanen J, Lassila A. Cumulative inaccuracies in implementation of additive manufacturing through medical imaging, 3D thresholding, and 3D modeling: a case study for an end-use implant. Appl Sci. 2020;10:2968. 10.3390/app10082968.

[30] Ogden KM, Aslan C, Ordway N, Diallo D, Tillapaugh-Fay G, Soman P. Factors Affecting Dimensional Accuracy of 3-D Printed Anatomical Structures Derived from CT Data. J Digit Imaging. 2015;28:654–63. 10.1007/s10278-015-9803-7. 25982877 10.1007/s10278-015-9803-7 PMC4636725

[31] materialise, CT SCAN PROTOCOL: Acetabular Tumor Reconstruction. https://assets-eu-01.kc-usercontent.com/8ff24b0e-57a3-0157-62d1-fa4ac9734eb5/4732d992-5ce3-4970-b296-d0fcb2b96dc2/CT%20Scan%20Protocol%20-%20aMace%20Onco%20-%20English%20-%20%20L-101698-02.pdf (accessed 20 April 2024).

[32] Dzierżak R, Omiotek Z, Tkacz E, Uhlig S. Comparison of the classification results accuracy for CT soft tissue and bone reconstructions in detecting the porosity of a spongy tissue. J Clin Med. 2022;11:4526. 10.3390/jcm11154526. 35956142 10.3390/jcm11154526 PMC9369728

[33] Giambini H, Dragomir-Daescu D, Huddleston PM, Camp JJ, An K-N, Nassr A. The effect of quantitative computed tomography acquisition protocols on bone mineral density estimation. J Biomech Eng. 2015;137;114502.10.1115/1.4031572 26355694 10.1115/1.4031572 PMC4844109

[34] Jiang C, Jin D, Ni M, Zhang Y, Yuan H. Influence of image reconstruction kernel on computed tomography-based finite element analysis in the clinical opportunistic screening of osteoporosis-A preliminary result. Front Endocrinol (Lausanne). 2023;14:1076990. 10.3389/fendo.2023.1076990. 36936156 10.3389/fendo.2023.1076990 PMC10014549

[35] Neubauer J, Wilhelm K, Gratzke C, Bamberg F, Reisert M, Kellner E. Effect of surface-partial-volume correction and adaptive threshold on segmentation of uroliths in computed tomography. PLoS ONE. 2023;18: e0286016. 10.1371/journal.pone.0286016. 37352326 10.1371/journal.pone.0286016 PMC10289361

[36] Fogarasi M, Coburn JC, Ripley B. Algorithms used in medical image segmentation for 3D printing and how to understand and quantify their performance, 3D Print. Med. 2022;8:18. 10.1186/s41205-022-00145-9. 10.1186/s41205-022-00145-9 PMC9229760 35748984

[37] Ramian J, Ramian J, Dziob D. Thermal deformations of thermoplast during 3D printing: warping in the case of ABS. Materials (Basel). 2021;14:7070. 10.3390/ma14227070. 34832469 10.3390/ma14227070 PMC8620654

[38] Brion DAJ, Pattinson SW. Generalisable 3D printing error detection and correction via multi-head neural networks. Nat Commun 13. 2022;13:4654. 10.1038/s41467-022-31985-y. 35970824 10.1038/s41467-022-31985-y PMC9378646

[39] International Organization for Standardization, ISO/ASTM 52920:2023 Additive manufacturing - Qualification principles - Requirements for industrial additive manufacturing processes and production sites., 1st ed. 25.030, 2023.

[40] Engelkes K. Accuracy of bone segmentation and surface generation strategies analyzed by using synthetic CT volumes. J Anat. 2021;238:1456–71. 10.1111/joa.13383. 33325545 10.1111/joa.13383 PMC8128766

[41] Kascenas A, Sanchez P, Schrempf P, Wang C, Clackett W, Mikhael SS, Voisey JP, Goatman K, Weir A, Pugeault N, Tsaftaris SA, O’Neil AQ. The role of noise in denoising models for anomaly detection in medical images. Med Image Anal. 2023;90: 102963. 10.1016/j.media.2023.102963. 37769551 10.1016/j.media.2023.102963

